# Demographic characteristics and comorbidities in patients with oral mucous membrane pemphigoid and pemphigus

**DOI:** 10.1007/s00784-026-07228-5

**Published:** 2026-10-21

**Authors:** Lili Fan, Jaana Willberg, Johanna Uittamo, Auli Suominen, Hanna K. Laine

**Affiliations:** 1 Department of Oral Pathology and Oral Radiology, Institute of Dentistry, University of Turku, Lemminkäisenkatu 2, Turku, 20520 Finland; 2 Department of Pathology, TYKS Laboratories, Turku University Hospital, Turku, Finland; 3 Department of Oral and Maxillofacial Diseases, Turku University Hospital, Turku, Finland; 4 Department of Oral and Maxillofacial Diseases, Helsinki University Hospital and University of Helsinki, Helsinki, Finland; 5 Department of Community Dentistry, Institute of Dentistry, University of Turku, Turku, Finland

**Keywords:** Autoimmune blistering disease, Oral cavity, Oral mucosal disease, Mucous membrane pemphigoid, Pemphigus vulgaris

## Abstract

**Objectives:**

Oral autoimmune blistering diseases (AIBDs) are a group of rare, chronic disorders causing different symptoms on the oral mucosa. The most common oral AIBDs are mucous membrane pemphigoid (MMP) and pemphigus vulgaris (PV). The aim of this study was to describe the demographic and clinical characteristics, as well as comorbidities, of patients with oral AIBDs in Southern and Western Finland.

**Materials and methods:**

This retrospective two-center study included patients diagnosed with AIBD with oral symptoms treated at Helsinki University Hospital and Turku University Hospital between the years 2000 and 2022. Statistical analyses included descriptive statistics, t-tests, Chi-square tests, Fisher’s exact tests, and likelihood ratio tests.

**Results:**

Of the 115 patients (65.2% women, mean age at diagnosis 66 years) analyzed, 86.1% were diagnosed with MMP. Patients with PV were significantly younger than patients with MMP. Most of the patients who had available lifestyle information reported being non-smokers and non-alcohol users. Approximately one third of the patients had an autoimmune or neurological comorbidity.

**Conclusions:**

There is a female predominance in oral AIBDs. Patients with MMP were older at diagnosis than patients with PV (*p* < 0.001). Comorbidity comparisons between diagnostic groups were limited by the small PV subgroup and should be interpreted as descriptive.

**Clinical relevance:**

MMP and PV are rare chronic AIBDs that can cause painful oral symptoms and thus impair quality of life. With an aging population and increasing immigration, the prevalence of these rare diseases in Western countries including Finland may rise. Therefore, early recognition and diagnosis with proper treatment and follow-up is needed.

## Introduction

Oral autoimmune blistering diseases (AIBDs) are a group of rare, chronic disorders that can cause different symptoms on the oral mucosa such as desquamative gingivitis, blistering, and ulcers [1]. The most common oral AIBDs are mucous membrane pemphigoid (MMP) and pemphigus vulgaris (PV), both of which can affect the oral mucosa causing significant pain, ulceration, and difficulties in eating and speaking, and thus impairing quality of life [2, 3]. Oral AIBDs may present exclusively in the oral cavity without skin lesions, highlighting the importance of early recognition by oral health specialists [1, 4, 5]. Furthermore, initial symptoms of AIBDs often present in the oral mucosa, especially in PV [1].

AIBDs are divided into two main categories, pemphigoid and pemphigus diseases, based on the location of the blistering in the epithelium of skin or mucosa. In pemphigoid diseases, such as MMP, the blistering occurs subepithelially, whereas in pemphigus diseases, including PV, the separation takes place intraepithelially [6, 7].

The estimated annual incidence of MMP is two new cases per million per year, predominantly affecting women aged over 60 [8, 9]. PV’s reported incidence globally is 2.83 cases per million per year with the mean age at diagnosis of 36.5–71 years. PV is reported more frequently in certain ethnic groups [2, 10, 11]. The etiology of both diseases remains unclear, however, there is evidence suggesting that PV may be triggered by external factors such as stress, medications, allergens, and viruses, whereas both PV and MMP may have genetic associations [2, 12]. Both diseases are chronic, often requiring long-term topical and/or systemic immunosuppressive therapy and multidisciplinary follow-up [6, 12].

Initial symptom of MMP may occur on the oral mucosa with the gingiva being the most common location [1, 3, 5]. Typically, PV first manifests on the oral mucosa followed later by skin involvement [1, 2, 12]. Desquamative gingivitis is the most common clinical feature in patients with MMP but can occur also in PV [3, 4]. Other oral features of MMP and PV include pain, soreness, erosions, bleeding, and peeling of the mucosa [1, 4]. The oral manifestations of AIBDs can resemble other oral diseases such as oral lichen planus, oral candidiasis, and herpetic gingival stomatitis, complicating differential diagnosis [7, 12]. The diagnosis of oral AIBDs is based on clinical assessment of oral mucosal lesions, confirmed by histopathology and direct immunofluorescence testing [1, 7, 12, 13].

Earlier studies have suggested possible associations between AIBDs and neurological diseases, such as Alzheimer’s disease as well as other immune-mediated diseases, potentially through shared autoimmune pathways [14–17]. With Finland’s aging population and increasing diversity, the burden of oral AIBDs may rise, highlighting the need for national data on epidemiology, clinical characteristics, and comorbidities.

Despite international studies, there are currently no published Finnish or Northern European cohort studies focusing on oral AIBDs. Therefore, the aim of this descriptive, retrospective two-centre study was to characterize the demographics, clinical features, and comorbidities of patients with oral AIBDs treated at Western and Southern Finland between 2000 and 2022.

## Materials and methods

We collected patients with oral AIBD diagnosed and treated between 2000 and 2022 at the Helsinki University Hospital, Helsinki, Finland or Turku University Hospital, Turku, Finland. These tertiary-care academic centres represent 1.78 million [18] inhabitants in Helsinki and Uusimaa and 0.5 million [19] in the region of Southwest Finland respectively, covering approximately one-third of the Finnish population.

The initial database search identified 226 patients, of whom 182 were from the Helsinki and Uusimaa region and 44 from the region of Southwest Finland respectively. The search only included patients aged 18 years or older and was based on ICD-10 codes L12.0 - L12.9 for pemphigoid and L10.0 - L10.9 for pemphigus. To form the final study cohort, we manually reviewed all these patients. The inclusion criteria were a confirmed diagnosis of an AIBD, established with histopathological findings, direct immunofluorescence and/or serological evidence as well as documented oral involvement. No other pemphigus subtypes fulfilled our inclusion criteria but L10.0 for PV and L10.1 for pemphigus vegetans. Pemphigus vegetans is generally considered a rare clinical variant of pemphigus vulgaris and accounts for approximately 1–2% of all pemphigus cases. Therefore, patients with pemphigus vulgaris and pemphigus vegetans were analyzed as a single group and are hereafter collectively referred to as pemphigus vulgaris (PV) [20].

The diagnosis in Finland is made based on the recommendations of the European guidelines [13, 21]. When AIBD is suspected based on clinical characteristics or histopathological findings, the diagnosis requires a confirmation by a positive immunofluorescence (IF) test. In addition, serological testing is used to support the diagnosis. From the final study cohort, we collected data on patient characteristics, laboratory analysis, histological parameters, treatment modalities, comorbidities, and follow up reviewing medical records.

Finnish Social and Health Data Permit Authority Findata approved the study concept (THL/3815). Patient data were anonymized prior to analysis.

Statistical analyses were performed using IBM SPSS statistics (version 27). Descriptive statistics were calculated for all variables to summarize patient characteristics. Associations between categorical variables were analysed using likelihood ratio tests, Chi-square test, or when assumptions were failed with Fisher’s exact test. Differences in continuous variables were evaluated using independent-samples t-tests. Associations between comorbidities and disease group were assessed in both the complete dataset and in analyses stratified by age into three age groups: 20–49, 50–69, and 70–99 years. A p-value less than 0.05 was considered statistically significant. For variables with missing data, percentages were calculated using available-case analysis based on patients with documented information.

## Results

### Patient characteristics

Table 1 shows the main characteristics of 99 patients with oral MMP and 16 patients with oral PV. Over half (51.3%) of the patients were diagnosed in oral and maxillofacial departments, 38.3% were diagnosed in dermatological departments and two patients (1.7%) were diagnosed in ear, nose and throat departments. Of the 115 patients, 65.2% were women, and the mean age at diagnosis was 66 years. Among patients who reported lifestyle information, 80.4% were non-smokers, and 59.1% were non-alcohol users. A total of three patients had missing or unreported information regarding tobacco use, and a total of 27 patients had missing or unreported information regarding alcohol use. Smoking or alcohol use did not associate significantly with the diagnosis of AIBD. Most patients (75.7%) were treated at Helsinki University Hospital and 73% of patients were alive at the end of the study’s follow-up period (2022).

**Table 1:** Characteristics of 115 patients with oral autoimmune blistering diseases

	Total (*n*/%)	Pemphigoid (*n*/%)	Pemphigus (*n*/%)
Sex			
Men	40/(34.8%)	33/(33.3%)	7/(43.8%)
Women	75/(65.2%)	66/(66.7%)	9/(56.3%)
Total	115/(100%)	99/(100.0%)	16/(100.0%)
Hospital department at diagnosis			
Oral and maxillofacial diseases	59/(51.3%)		
Dermatological diseases	44/(38.3%)		
Ear, nose and throat diseases	2/(1.7%)		
Age at diagnosis			
20–29	4/(3.5%)	1/(1.0%)	3/(18.8%)
30–39	2/(1.7%)		2/(12.5%)
40–49	9/(7.8%)	5/(5.1%)	4/(25.0%)
50–59	17/(14.8%)	14/(14.1%)	3/(18.8%)
60–69	29/(25.2%)	29/(29.3%)	
70–79	30/(26.1%)	27/(27.3%)	3/(18.8%)
80–89	19/(16.5%)	18/(18.2%)	1/(6.3%)
90–99	3/(2.6%)	3/(3.0%)	
Smoking			
No	90/(80.4%)	75/(78.1%)	15/(93.8%)
Yes	5/(4.5%)	5/(5.2%)	
Former-smoker	17/ (15.2%)	16/(16.7%)	1/(6.3%)
Alcohol use			
No	52/(59.1%)	45/(58.4%)	7/(64%)
Yes	34/(38.6%)	31/(40.3%)	3/(27.3%)
Quit	2/(2.3%)	1/(1.3%)	1/(9.1%)
Alive			
Yes	84/(73%)	69/(69.7%)	15/(93.8%)
No	31/(27%)	30/(30.3%)	1/(6.3%)
Treatment			
Non-pharmacological treatment	1/(0.9%)	1/(1.0%)	
Topical medication	31/(27%)	29/(29.3%)	2/(12.5%)
Systemic medication	9/(7.8%)	4/(4.0%)	5/(31.3%)
Topical + systemic medication	74/(64.3%)	65/(65.7%)	9/(56.3%)

A total of 86.1% of patients were diagnosed with MMP, of whom 66.7% were women and the F: M ratio was 2:1. Among patients diagnosed with PV, 56.3% were women and the F: M ratio was 9:7. Gender did not associate significantly with the diagnosis. The mean age at diagnosis for MMP was 69.3 years (range, 29.0–93.9 years). The mean age at diagnosis for PV was 48.7 years (range, 24.4–80.4). The mean ages at the diagnosis for MMP and PV differed (*p* < 0.001). The most common age at diagnosis (mode) for patients with MMP was 75 years. The mode age in PV patients was bimodal, with the most common ages at diagnosis being 26 and 40 years.

### Comorbidities

Thirty-two patients with MMP or PV had other autoimmune or neurological diseases (Table 2). Among these patients, the most common recorded comorbidity was Alzheimer’s disease (7.0%). No statistically significant associations between disease group and the comorbidities grouped into rheumatic diseases, bowel diseases, mucocutaneous diseases, or neurological diseases were observed in the complete dataset or in the age groups 20–49, 50–69, and 70–99 years (*p* > 0.05). None of the patients had known relatives with a diagnosis of AIBD.

**Table 2:** Characteristics of comorbidities of 32 patients with oral mucous membrane pemphigoid or oral pemphigus vulgaris

	Total *n*/(%)	Pemphigoid *n*/(%)	Pemphigus *n*/(%)
Autoimmune disease			
Rheumatoid diseases*	4/(3.5%)	4/(4%)	
Bowel diseases**	4/(3.5%)	3/(3.0%)	1/(6.3%)
Mucocutaneus diseases ***	4/(3.5%)	4/(4%)	
Sjögren’s syndrome	1/(0.9%)	1/(1.0%)	
Hypothyreosis	4/(3.5%)	3/(3.0%)	1/(6.3%)
Neurological disease			
Alzheimer’s disease	8/(7%)	8/(8.1%)	
Vascular dementia	2/(1.7%)	2/(2.0%)	
Progressive vascular cognitive disorder	1/(0.9%)	1/(1.0%)	
Dementia	4/(3.5%)	4/(4.0%)	
Parkinson’s disease	3 (2.61%)	3 (3.0%)	

* Rheumatoid arthritis, Psoriatic arthritis, Seronegative rheumatoid arthritis, Seropositive rheumatoid arthritis, Ankylosing spondylitis

**Crohn’s disease, Ulcerative colitis, Celiac disease

*** Lichen sclerosus, Lichen planus, plasma cell vulvitis

## Discussion

This study analyzed 115 patients with oral AIBDs treated in Helsinki and Turku, Finland, between the years of 2000–2022, focusing on demographic characteristics and comorbidities. Two-third of the patients were women and 86.1% suffered from MMP. Patients with PV were significantly younger than patients with MMP. Patients who had available lifestyle information were typically non-smokers and non-alcohol users. Approximately one third of the patients had an autoimmune or neurological comorbidity, although no significant associations between comorbidities and diagnosis were identified.

Most diagnoses in this study were confirmed in oral and maxillofacial departments, highlighting the importance of recognizing oral symptoms of AIBDs, especially the role of dentists and other oral health specialists. The diagnosis of oral AIBDs requires specific expertise of the oral mucosal diseases and the diagnostic procedures used in tertiary care. In addition, the treatment and follow-up of these diseases are usually also conducted in tertiary care.

In this study, the proportion of oral MMP was 86.1% of all cases. Although, no earlier studies specifically reported the prevalence of oral AIBDs, previous studies have described oral involvement in up to 90% of PV patients but exclusively oral involvement without skin lesions in only about 50% of cases. In contrast, oral involvement in MMP is described as the primary clinical manifestation in up to 85% of patients [6, 22–24]. This difference likely reflects the mucosa dominant nature of MMP, and the study focus on oral disease, as patients with exclusively oral symptoms are more likely to be identified and managed by oral healthcare specialists.

The lower proportion of PV observed in this study may be partly explained by ICD-10 diagnostic coding practices. Suspected PV is sometimes initially coded as L30.9 (unspecified dermatitis), which was not included in our database search that focused on confirmed pemphigus and pemphigoid ICD-10 codes. This may have led to exclusion of some early phase PV cases.

Most patients were female, which aligns with the previous studies of female predominance of AIBDs [1, 3, 4]. The mean age at diagnosis in MMP was 69.3 years and PV 48.7 years with a statistically significant difference. These findings are consistent with earlier epidemiological reports that describe MMP as a disease of older population [9], whereas PV typically manifests earlier in life with previous studies reporting onset in patients as young as their mid-twenties [11, 25–27].

Although oral involvement was documented, the specific intraoral sites affected were not recorded in our dataset. This limits comparison with previous literature, which shows that in MMP the gingiva is the most frequently affected site and often presenting as desquamative gingivitis while PV commonly begins on the oral mucosa, especially on the buccal mucosa, palate, or gingiva [1, 2, 4].

In the current study, among those who reported alcohol use, 59.1% stated no alcohol use and 2.3% stated that they had stopped their use of alcohol. This proportion is substantially higher than in the Finnish adult population as according to the Finnish Institute for Health and Welfare (THL) approximately 12% of adults were non-drinkers in 2023 [28]. However, alcohol use in this study was based on patients´ self-report, and no standardized definitions or quantities were recorded in the medical records The retrospective study design may also limit the accuracy of alcohol use as documentation practices vary, and alcohol use may not have been consistently recorded during clinical visits.

Earlier studies are in line with our findings regarding alcohol and tobacco use. Patients with autoimmune blistering diseases tend to report lower rates of smoking and alcohol consumption with the general population. In a study on pemphigus epidemiology, smoking prevalence was consistently lower among pemphigus patients across multiple cohorts [29]. Likewise, studies evaluating environmental and lifestyle factors in autoimmune blistering diseases have not identified increased alcohol use among patients, but rather, rates of alcohol consumption are generally equal to or lower than those in matched populations [30].

AIBDs have been related to chronic autoimmune or neurological conditions [14–17]. For example, autoimmune conditions such as rheumatoid arthritis and autoimmune thyroid disease have been reported among patients with AIBDs [16, 17], while Alzheimer’s disease has been identified as a frequent neurological comorbidity in older adults with pemphigus or pemphigoid [14, 15]. In the current study, approximately one-third of the patients had autoimmune or neurological diseases, the most frequent being Alzheimer’s disease. In the general Finnish adult population Alzheimer’s disease and other dementias affect around 150 000 of individuals with estimated 23 000 new diagnoses made annually and prevalence increasing with age [31]. Given the advanced age of many patients in this study, particularly those with MMP, the observed occurrence of Alzheimer’s disease may partly reflect age-related background risk. Although, no significant statistical association was found between comorbidities and diagnosis, these observations are consistent with earlier findings that AIBDs may coexist with other chronic autoimmune or neurological conditions [14–17]. Earlier epidemiological studies have shown that people with bullous pemphigoid have a higher risk of developing dementia, including Alzheimer’s disease, suggesting a possible connection between skin autoimmunity and changes in the nervous system [14, 32].

The strengths of this study include a long follow-up period of up to 22 years and the inclusion of two large university hospitals in Finland, thereby covering a large proportion of the Finnish population and capturing all cases of confirmed oral AIBDs during the follow-up period. The main limitation of this study is the relatively small study population size due to the rarity of these diseases in Finland and the focus of the study’s oral involvement only.

## Conclusions

In this study most diagnoses were made and confirmed in oral and maxillofacial departments, which highlights the role of oral health specialists in the identification and management of AIBDs. There was a female predominance in oral AIBDs and MMP patients were significantly older at the time of diagnosis than patients with PV. Regarding lifestyle factors, AIBD-patients with available documentation were typically non-alcohol users and non-smokers. No significant associations between comorbidities and diagnosis were identified in this study. However, the limited number of PV patients may have reduced the ability to detect potential associations; therefore, these findings should be interpreted as descriptive.

## Data Availability

The data that support the findings of this study were collected from Helsinki University Hospital and Turku University Hospital. Due to ethical and legal restrictions related to patient confidentiality, the data are not publicly available. Data may be available from the corresponding author upon reasonable request and with permission from the respective institutions.
